# Molecular docking analysis of COX-2 with compounds from Piper longum

**DOI:** 10.6026/97320630017623

**Published:** 2021-06-30

**Authors:** Dhirendra Tripathi, Sravanthi Koora, K Satyanarayana, S Saleem Basha, Selvaraj Jayaraman

**Affiliations:** 1 Department of Otorhinolaryngology, Government Medical College, Shivpuri, Shivpuri - 473638; 2 Department of Pharmacology, Government Medical College Siddipet 502103, Siddipet, Telangana; 3 Department of Biochemistry, Government Medical College Siddipet,Siddipet 502103, Telangana India; 4 Department of Medical Biochemistry, School of Medicine, Haramaya university, Harar Campus,Ethiopia; 5 Department of Biochemistry, Saveetha Dental College and Hospitals, Saveetha Institute of Medical and Technical Sciences,Chennai-600 077, Indi

**Keywords:** Anti-inflammatory compunds, COX-2, Piper longum, molecular docking

## Abstract

Piper longum (Indian long pepper) is known for its use as an anti inflammatory agent in Indian Ayurvedic System of medicine. Therefore, it is of interest to document the molecular docking analysis of compounds from Piper longum with COX-2 using the Autodock Vina PyRx tool. Molecular docking results show that asarinine, sesamine, fargesin, and piperlonguminine have optimal binding energy of 10, 10, -9.5 and 9.4 Kcal/mol, respectively for further consideration.

## Background

The inflammatory reactions linked with the release of histamine,bradykinin & prostaglandins [1] are part of the host defence mechanisms. COX-1 is necessary for the creation of important biological mediators like prostanoids, including prostaglandins, prostacycline and thromboxane, which are involved in causing pain, blood clotting and stomach protection [2]. COX-2 is involved in inflammatory pain and plays a significant role in the biosynthesis of prostaglandin in inflammatory cells [3 - check with author]. COX-2 is typically specific to inflamed tissue [4]. Several COX-2 inhibitors like celecoxib and rofecoxib are known [5]. Coxib medicines such as rofecoxib (Vioxx®) and valdecoxib (Bextra®) were withdrawn due to increased risk of long-term heart attacks and strokes [6, 7]. Hence, the need to develop effective inhibitors to COX-2 from natural sources is highly imperative. Piper longum linn [8] (piperaceae) is a commonly available tropical climbing shrub throughout India. Piper longum (Indian long pepper) is known for its use as an anti inflammatory agent in Indian Ayurvedic System of medicine [9-11]. Therefore, it is of interest to document the molecular docking analysis of compounds from Piper longum with COX-2 using the Autodock Vina PyRx tool.

## Materials and Methods:

### Protein preparation:

The X-ray crystallographic structure of the protein COX-2 (PDB ID: 5IKT) at a resolution of 3.0Å was downloaded from the Protein Data Bank. Water molecules, ligands, and other heteroatoms are deleted. The addition of hydrogen atoms to the protein was completed using the CHARMm force field. Energy minimization was completed using the conjugate gradient method with an RMS gradient of 0.01kcal/Å mol in Accelyrs Discovery studio client software (version 2.5).

### Ligand preparation:

22 structures of ligand molecules (Table 1 - see PDF) were downloaded from the pubchem database. Accelyrs Discovery studio client (version 2.5) software was used for energy minimization.

### Molecular docking

Molecular docking was completed using the Autodock Vina PyRx program using standard procedures [12]. The interactions of complex protein-ligand conformations were analyzed using PyMol.

## Results and Discussion:

**Figure 1:**
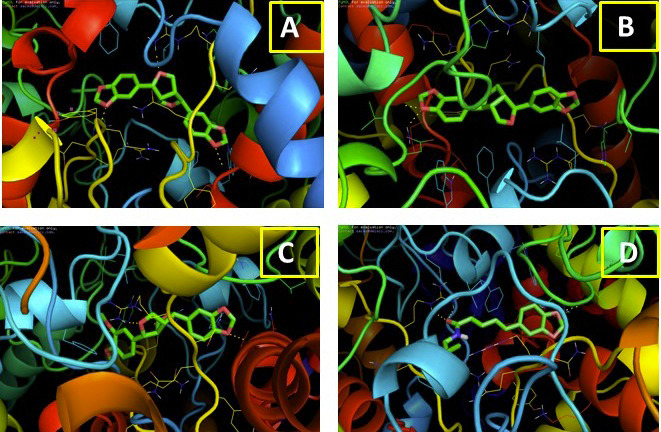
Molecular interaction of COX-2 with (a) asarinine; b) sesamin; (c) Fargesin and (d) Piperlonguminine

It is of interest to document the molecular docking analysis [13-14] of compounds from Piper longum with COX-2 using the Autodock Vina PyRx tool. Data shows that 4 compounds showed good binding energy (Table 2 - see PDF). The binding energies are -10, -10, -9.5,and -9.4 kcal/mol for asarinine, sesamin, fargesin and piperlonguminine, respectively. The interaction energies for asarinine and sesaminthe (Figure 1) into the COX-2 active site are greater than the other two compounds. Asarinine formed three hydrogen bonds interaction through the amino acids ASN-375, ARG-376 and VAL-538 at a distance of 2.2, 2.3 and 2.4 Å, respectively. Sesamin formed the two hydrogen bond interactions with VAL-228, ASN-537 at a distance of 2.2, and 2.4 Å. Fargesin have a binding energy of -9.5 with two hydrogen bond interactions with the amino acids of ARG- 376, VAL-538 at distance of 2.1 and 2.5 Å. The piper longuminine have a binding energy of -9.4 kcal/mol and formed three hydrogen bond interactions at distance of 2.6, 2.7 and 2.4 A through the amino acid residues VAL-228, ARG-376 and ASN-537. Analysis shows that these compounds have hydrogen bonding with the residue ARG-376 for further consideration.

## Conclusion

We show the optimal binding features of compounds () from Piper longum with COX-2 for further consideration in the context of inflammation.
